# The National Security Implications of HIV/AIDS

**DOI:** 10.1371/journal.pmed.0030171

**Published:** 2006-06-13

**Authors:** Harley Feldbaum, Kelley Lee, Preeti Patel

## Abstract

Feldbaum and colleagues look at evidence on the links between HIV and national security, and evaluate the risks and benefits of addressing HIV/AIDS as a national security issue.

Two leaders in the global fight against HIV/AIDS—Richard Feachem, Executive Director of the Global Fund to Fight AIDS, Tuberculosis, and Malaria, and Peter Piot, Executive Director of the Joint United Nations Programme on HIV/AIDS (UNAIDS)—have recently argued that HIV represents an extraordinary national security threat [ 1, 2]. Their intent has been to appeal to the material and strategic interests of states in order to increase high-level commitment to the fight against HIV/AIDS. While increased engagement by political leaders is clearly welcome, a public health perspective on the linkage between public health and national security has so far been missing. The benefits and dangers of justifying efforts to address the pandemic in terms of its impact on national security are underappreciated.

Despite the high-profile linking of HIV/AIDS and security, including four United Nations Security Council (UNSC) meetings and prominent mention within the United States national security strategy, critical debate about the ways in which public health interacts with the security interests of states are scarce in public health journals. Journals have examined the ways national security issues, including the recent war in Iraq and the Israeli–Palestinian conflict [ 3, 4], have negatively affected public health. However, the ways public health affects national security interests have rarely been considered. It is essential to examine this debate, including evidence for the links between HIV/ AIDS and national security, from a public health perspective because of the implications this linkage has for the direction and funding of global HIV/ AIDS efforts.

This paper argues for greater understanding and analysis of the public health–national security nexus to ensure this linkage benefits the fight against HIV/AIDS. Successfully negotiating this nexus is essential because the humanitarian objectives of global health do not fit easily into the state-centered perspective of national security. Global health works to improve the health of all people within and across states, while the national security field works to protect the people, property, and interests of only one state. This article will present the unique national security perspective on the HIV/AIDS pandemic and evidence on the links between HIV/AIDS and national security, and will evaluate the risks and benefits of addressing HIV/ AIDS as a national security issue.

## The National Security Perspective on the HIV/AIDS Pandemic

National security is traditionally defined as the protection of a state's territory, population, and interests against external threats. While recognizing the humanitarian dimensions of the HIV/AIDS pandemic, national security analyses rely on the human impacts of the disease to be large enough to affect the military, political, and economic interests of a state. For example, a partially declassified US National Intelligence Council estimate argues that infectious diseases threaten American national security because they will “endanger US citizens at home and abroad, threaten US armed forces deployed overseas, and exacerbate social and political instability in key countries and regions in which the United States has significant interests” [ 5]. This focus on strategic interests within national security thinking partially explains why other global health problems of comparably high morbidity and mortality, such as noncommunicable diseases, are not currently considered threats to national security. The effects of these global health problems are not seen to significantly impact the strategic interests of powerful states.

Three main arguments linking HIV/ AIDS and national security emerge from the security community's analyses of the HIV/AIDS pandemic. The first describes the impact of HIV/AIDS on individuals critical to the maintenance of state and international security: soldiers and peacekeepers. The second argument suggests that the epidemic in some sub-Saharan African nations may cause state instability and failure. The third argument focuses on the security effects of the worsening pandemic on the large, strategically important states of Russia, India, and China (see Figure 1). The evidence for each of these arguments is considered in turn.

**Figure 1:**
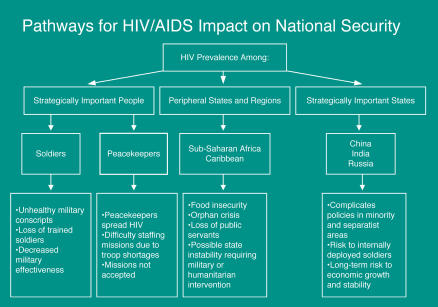
Pathways through Which HIV/AIDS Has an Impact upon National Security

## The Impact of HIV/AIDS on Strategically Important Populations

### Soldiers

HIV/AIDS is severely affecting the armed forces of many countries. Accurate data on prevalence of HIV among soldiers is difficult to obtain because affected states either do not collect or do not want HIV prevalence data published. In 1998, UNAIDS estimated that sexually transmitted disease “rates among armed forces are generally 2 to 5 times higher than in civilian populations” [ 6]. Recent estimates are more conservative and suggest that HIV prevalence among armed forces is equal to or slightly greater than civilian rates from the same country [ 7]. Despite the lack of reliable data, there is evidence that the disease is affecting African militaries. Ugboga Nwokoji and Ademola Ajuwon state that “AIDS is now the leading cause of death in military and police forces in some African countries, accounting for more than half of in-service mortality” [ 8]. The US National Intelligence Council estimates that “HIV/AIDS probably will complicate staffing in the military officer corps” of Nigeria and Ethiopia [ 5].

However, the impact of HIV/AIDS on militaries is not limited to Africa. In Russia, 9,000 potential draftees have been rejected for service because of testing HIV positive in the last five years, with 5,000 rejected in the last two years alone (M. Feshbach, unpublished data). While China and India's large populations provide some insulation against shortages in military personnel, these countries are increasingly monitoring AIDS in their militaries as epidemics in these countries spread [ 5].

The impact that high HIV prevalence will have on the strategic capabilities of militaries is complex. Most analysts emphasize the negative security implications of increasing rates of HIV infection in militaries. The loss of highly trained, professional soldiers to AIDS will have a major impact on affected armed forces. Trained soldiers are difficult and expensive to replace, and their absence interrupts the training of younger recruits. Armed forces that rely wholly or partially on conscripts face a decreasing pool of healthy recruits as HIV/AIDS continues to spread. Russia's HIV epidemic is already exacerbating an existing shortage of healthy individuals available for military service. It is also argued that armed forces with high HIV prevalence may incur mounting costs to treat soldiers with antiretroviral drugs, leading the military to seek greater proportions of public expenditure while rendering them less able to protect national and international interests [ 9, 10].

It is important to note that some analyses conflate HIV and AIDS, assuming all soldiers who are HIV positive will not be able to perform their duties because of AIDS. Thus, the security implications of HIV may be less than initially perceived, especially for militaries relying on conscription, because many soldiers who are HIV positive will have completed their duty by the time they develop symptoms of AIDS. Also, contrary to arguments that HIV will worsen national security is the idea that higher rates of HIV among militaries could have a beneficial strategic effect by constraining “offensive military plans in bellicose countries” [ 9]. Decreased military effectiveness may make some countries more likely to turn to nonmilitary means to resolve conflicts and promote their interests. However, there is no available evidence to date that HIV has inspired or foreclosed armed conflict. The strategic impact of high HIV prevalence on the armed forces remains complex and dependent upon other country-specific factors.

### Peacekeepers

Concern about peacekeepers spreading HIV/AIDS while on missions brought the pandemic to the attention of the UNSC in 2000. Then US Ambassador to the UN Richard Holbrooke argued that it was a cruel irony to send peacekeepers to stop conflict only to find that they have unintentionally spread HIV [ 11]. Fears that peacekeepers continue to spread HIV have been reinforced by recent accusations of sexual abuse perpetrated by peacekeepers while deployed in the Democratic Republic of Congo [ 12]. While these acts may have minimal impact on the regional AIDS epidemic, they seriously undermine trust in UN peacekeeping missions.

In addition to peacekeepers directly spreading HIV, high rates of HIV among the militaries in troop-contributing countries may make it more difficult to staff peacekeeping missions [ 9, 13]. High rates of HIV in the South African and Nigerian militaries in particular, which are major contributors of peacekeeping troops, may imperil African-led responses to regional crises such as that in Sudan. Countries may also be less willing to contribute personnel for peacekeeping operations if soldiers risk returning from the mission infected with HIV [ 9]. Among Nigerian peacekeepers in Sierra Leone, HIV prevalence increased with the length of duty, from 7% to 10% to 15% over three years (A. Adefolalu, unpublished data). If peacekeeping tours of duty continue to be correlated with increasing HIV infections, countries may begin to limit the amount of time they commit troops to peacekeeping missions.

A final complication is that countries may object to hosting peacekeepers that come from countries with high HIV/AIDS prevalence [ 9]. In 2001, Eritrea unsuccessfully demanded that the UNSC prevent troops who were HIV positive from being deployed in peacekeeping operations on the border of Ethiopia and Eritrea [ 14]. Overall, there is growing evidence that the HIV/AIDS pandemic poses increasing challenges for the conduct of peacekeeping operations given the spread of HIV by peacekeepers, the reduced ability of countries to contribute peacekeepers, and the decrease in willingness of some countries to accept peacekeepers who may pose a disease risk to them.

## The Potential Effect of HIV/AIDS on State Stability

The effect of HIV/AIDS on state stability is perhaps the least studied, yet also the most feared, potential impact of the disease on security. The US National Intelligence Council argues that high rates of HIV/ AIDS are “likely to have significant economic, social, political, and military implications” in certain countries [ 15]. If these implications become severe, the AIDS epidemic could contribute to state instability.

### Sub-Saharan Africa

The repercussions of HIV affecting state stability differ depending upon the country and region affected. To date, sub-Saharan Africa has been the most highly affected region, and the pandemic is seen to represent a direct national security threat to countries in this region [ 16]. Despite life expectancy at birth falling to below 40 years in nine African nations, and 2.3 million deaths from AIDS in sub-Saharan Africa in 2004, there remains a lack of evidence directly linking HIV/AIDS and state instability [ 17]. Nonetheless, Peter Piot of UNAIDS warns, “How can governments function, public services operate, agriculture and industry thrive, and law enforcement and militaries maintain security, when they are being stripped of able-bodied and skilled women and men” [ 1]?

Evidence does exist that suggests AIDS is undermining the capacity of communities in Southern Africa to cope with food crises, producing a new variant of famine created by AIDS [ 18]. A number of authors argue that the growing number of children orphaned by AIDS poses significant risks to stability in highly affected states [ 19, 20], warning that the epidemic will “produce a huge and impoverished orphan cohort unable to cope and vulnerable to exploitation and radicalization” [ 5]. However, other evidence suggests that, while orphans are clearly disadvantaged, most are cared for by family members and are not turning to crime [ 21]. Because of the epidemic's gradual impact on the state, it may not cause destabilization alone, but may add to instability in sub-Saharan African countries already weakened by poverty and poor governance [ 9].

The consideration of sub-Saharan Africa on the Western security agenda is new. Before the events of 9/11, the huge human toll of HIV/AIDS in sub-Saharan Africa was insufficient to seriously engage Western security communities. The experience of 9/11, when terrorists attacked the US from a safe harbour within the failed state of Afghanistan, demonstrated the threat that failed states pose to powerful countries. The 2002 US national security strategy states clearly that “America is now threatened less by conquering states than we are by failing ones” [ 22]. The 2003 UK Ministry of Defence White Paper also prioritizes the risk of failed states [ 23]. While the role of HIV/AIDS in state failure remains unproven and is likely to be indirect, this perceived linkage has brought states and regions considered “peripheral” to Western security interests onto national security agendas.

## The Second Wave in Strategically Important States

The final argument linking HIV/AIDS and national security has focused on more traditional national security concerns by arguing that a “second wave” of HIV could destabilize powerful countries and regions critical to the US and, by extension, global strategic interests [ 15, 24]. The nations typically included in this group are Russia, India, and China. These are three of the seven declared nuclear states, and although the security impacts of HIV/ AIDS may be years away, increased instability in any of these countries would have major political, economic, and military repercussions.

Russia has rejected HIV positive individuals from military service.

Nicholas Eberstadt estimates that intermediate-to-severe epidemics in Russia, India, and China could account for between 193 and 259 million new cases of HIV by 2025— more than six times the global total of people living with HIV/AIDS today [ 25]. This second wave argument has been used to pressure these governments to more seriously address their growing HIV/ AIDS epidemics by appealing to their strategic interests. At the same time, it also demonstrates a potential distortion of prioritizing—based on perceived strategic importance rather than public health need.

From the perspective of the Russian, Indian, and Chinese governments, the link between HIV/AIDS and security raises internal security challenges. India and China have vast populations and small HIV epidemics as a proportion of the total population, making them less vulnerable than sub-Saharan Africa to shortages in workers and military recruits. Russia has rejected individuals who are HIV positive from military service since 2003, compounding its shortage of conscripts due to tuberculosis, drug use, alcoholism, and age demographics (M. Feshbach, unpublished data) [ 26]. The AIDS epidemic in all three countries has been driven in significant part by injection drug users along heroin trafficking routes, and their interactions in turn with sex workers and the general population [ 17, 27]. High HIV infection rates among injection drug users and their sexual partners in Xinjiang and Yunnan in China, and Manipur in India, overlap areas of ethnic minorities, political insurgency, and separatist sentiment. Xinjiang and Manipur have large numbers of Chinese and Indian security forces, respectively, who are at elevated risk of HIV infection through commercial sex with local populations. The Russian military faces similar, although smaller-scale, risks in Chechnya [ 28, 29].

This combination of drug trafficking and use, commercial sex, and growing HIV epidemics in separatist areas are major challenges for the Chinese, Indian, and Russian governments. Programs to prevent and control HIV/ AIDS in these regions may fail due to lack of trust, while doing nothing may result in further political tensions and even accusations of genocide [ 30]. China also faces political and economic fallout from HIV infections among its blood donors and “floating population.” Overall, the links between HIV/AIDS and strategically important countries and regions have received increased attention by Western governments and the governments of the countries concerned. There remain differences, however, in how these links are perceived, and the appropriate means of addressing the security impact of HIV/AIDS.

## The Risks and Benefits of Addressing HIV/AIDS as a National Security Threat

The available evidence to date on the links between HIV/AIDS and security suggests real, and potentially significant, risks to national, regional, and global security from the pandemic. In sub-Saharan Africa, the HIV/AIDS pandemic is already having a substantial impact on militaries and peacekeepers. In the strategically important states of Russia, India, and China, HIV/AIDS may contribute to medium- to long-term risks of state and regional instability. However, far more evidence is needed to support such claims.

What is clear is that arguments linking HIV/AIDS to national security have succeeded in elevating the disease to the highest levels of international politics, resulting in greater political commitment and funding. The chronic lack of resources to fight the disease and the neglect of the humanitarian crisis in sub-Saharan Africa seem to justify this strategic appeal to the “higher order” values of national interests and self-preservation that define the security community.

Nonetheless, it is also important to recognize that there are a number of potential risks in adopting a national security approach to fight HIV/AIDS. Countries classifying information on HIV/AIDS in their armed forces as national security secrets hinder the targeting, operation, and evaluation of HIV prevention and treatment programs for both soldiers and civilian populations that interact with them. A primary focus on the national security implications of the pandemic could cause an inappropriate redirection of HIV/AIDS resources toward strategically important countries or those supportive of the “War on Terror.” Conversely, a security community could conclude that higher rates of HIV/AIDS in certain militaries would actually benefit its own national interests because of a reduced ability by affected countries to launch offensive attacks. In Russia, India, and China, the security implications of HIV/AIDS involve vulnerable populations such as injection drug users, sex workers, and ethnic minorities in separatist areas. Addressing their health needs using a security-based rationale could lead to repression or increased stigmatization of persons living with HIV/AIDS, both counterproductive to effective public health practice. Finally, the security community seeking to win “hearts and minds” through health initiatives clouds the traditionally humanitarian role of public health and could lead to a loss of trust in the motives of public health professionals working on HIV/AIDS, an issue already fraught with sensitivities. The Soviet KGB disinformation campaign, suggesting that the US deliberately developed and spread HIV/AIDS, is an early example of the mixing of HIV/AIDS and security politics that still haunts public health activities today [ 31, 32].

Strengthening the evidence on the links between HIV/AIDS and national security is essential.

Successfully negotiating these risks is critical to ensuring that the public health–national security nexus benefits the fight against HIV/AIDS. Strengthening the evidence base on the links between HIV/AIDS and national security is essential. This includes better data on the epidemiology of HIV/AIDS among soldiers and peacekeepers, and the complex relationship between the disease and state stability. Equally important is the ability of the health community to appropriately harness the political clout and resources of the security community. Foremost is the need to demonstrate how proven principles and practices in public health must be maintained. The security community must be convinced of their value, notably a human rights–based approach, to national security interests. It is only with this starting point that the shared goal of effectively tackling the HIV/AIDS pandemic can be achieved together.

## 

**Figure:**
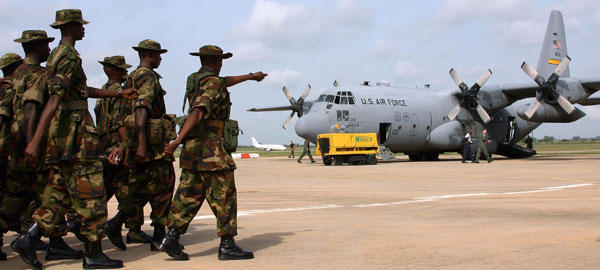
Nigerian troops prepare to be airlifted to Darfur, Sudan

## Abbreviations

UNAIDS: Joint United Nations Programme on HIV/AIDS
UNSC: United Nations Security Council
